# Europium sulfide nanoprobes predict antiretroviral drug delivery into HIV-1 cell and tissue reservoirs

**DOI:** 10.7150/ntno.59568

**Published:** 2021-04-27

**Authors:** Jonathan Herskovitz, Mahmudul Hasan, Jatin Machhi, Insiya Mukadam, Brendan M. Ottemann, James R. Hilaire, Christopher Woldstad, JoEllyn McMillan, Yutong Liu, Javier Seravalli, Anandakumar Sarella, Howard E. Gendelman, Bhavesh D. Kevadiya

**Affiliations:** 1 Department of Pathology and Microbiology, College of Medicine, University of Nebraska Medical Center, Omaha, NE 68198, USA.; 2 Department of Pharmacology and Experimental Neuroscience, University of Nebraska Medical Center, Omaha, NE 68198 USA.; 3 Department of Pharmaceutical Sciences, University of Nebraska Medical Center, Omaha, NE 68198 USA.; 4 Department of Otorhinolaryngology, University of Kansas Medical Center, Kansas City, KS 66213 USA.; 5 Brain Health Imaging Institute, Weill Cornell Medicine, New York, NY 10065 USA.; 6 Department of Radiology, University of Nebraska Medical Center, Omaha, NE 68198 USA.; 7 Department of Biochemistry, University of Nebraska Lincoln, Lincoln, NE 68588 USA.; 8 Nebraska Center for Materials and Nanoscience, University of Nebraska Lincoln, Lincoln, NE 68588 USA.

**Keywords:** HIV-1, antiretroviral, nanotheranostics, europium, molecular imaging, SPECT-CT

## Abstract

**Background:** Delivery of long-acting nanoformulated antiretroviral drugs (ARVs) to human immunodeficiency virus type one cell and tissue reservoirs underlies next generation antiretroviral therapeutics. Nanotheranostics, comprised of trackable nanoparticle adjuncts, can facilitate ARV delivery through real-time drug tracking made possible through bioimaging platforms.

**Methods:** To model HIV-1 therapeutic delivery, europium sulfide (EuS) nanoprobes were developed, characterized and then deployed to cells, tissues, and rodents. Tests were performed with nanoformulated rilpivirine (NRPV), a non-nucleoside reverse transcriptase inhibitor (NNRTI) used clinically to suppress or prevent HIV-1 infection. *First*, CD4+ T cells and monocyte-derived macrophages were EuS-treated with and without endocytic blockers to identify nanoprobe uptake into cells. *Second*, Balb/c mice were co-dosed with NRPV and EuS or lutetium^177^-doped EuS (^177^LuEuS) theranostic nanoparticles to assess NRPV biodistribution via mass spectrometry. *Third*, single photon emission computed tomography (SPECT-CT) and magnetic resonance imaging (MRI) bioimaging were used to determine nanotheranostic and NRPV anatomic redistribution over time.

**Results:** EuS nanoprobes and NRPV entered cells through dynamin-dependent pathways. SPECT-CT and MRI identified biodistribution patterns within the reticuloendothelial system for EuS that was coordinate with NRPV trafficking.

**Conclusions:** EuS nanoprobes parallel the uptake and biodistribution of NRPV. These data support their use in modeling NRPV delivery to improve treatment strategies.

## Introduction

Infection by the human immunodeficiency virus type 1 (HIV-1) requires lifelong daily adherence to antiretroviral therapy (ART). ART treatment suppresses viral replication, mitigates transmission, and delays acquired immunodeficiency syndrome (AIDS) onset. Lack of access to treatment, negative social stigma, and drug associated toxicities contribute to a nearly 41% regimen adherence failure rate 1, 2. Indeed, in the absence of ART, HIV-1 replicates continuously in virus target cells. These include CD4+ T lymphocytes and, to a lesser extent, mononuclear phagocytes (MP; monocytes, macrophages, and dendritic cells) promoting either long-lived cell reservoirs or selective cell death, respectively. Integration of proviral DNA in cells perpetuates infection, disease, and viral transmission. The means to improve antiretrovirals that maximally suppress HIV-1 replication or simplify pre-exposure prophylaxis (PrEP) drug regimens is of immediate need for viral therapy and prevention.

Recent clinical trials demonstrate the effectiveness of nanoformulated antiretrovirals as long-acting HIV-1 maintenance and prevention therapies. These are combinations of long-acting rilpivirine (RPV), a non-nucleoside reverse transcriptase inhibitor 3, and the integrase strand transfer inhibitor (INSTI), cabotegravir (CAB) 4. Once a month, or once every other month, dosing was demonstrated to be a non-inferior regimen to daily oral therapy 3-8. Similar strategies were recently developed for other antiretrovirals 9, 10. Indeed, encasing these drugs in polymer excipients facilitates a slow dissolution of drugs from intramuscular injection sites. Plasma drug levels of protease inhibitors (PI) ritonavir, indindavir, atazanavir (ATV) and the NNRTI, efavirenz, were raised 1.5-4 fold encased in poloxamer 188 excipients 11. Despite simplified dosing regimens, suboptimal delivery of INSTI to lymph nodes 12 and sequestration of nucleoside reverse transcriptase inhibitors by adipose tissues 13 prompt concern for low-level viral replication in tissues despite maintaining undetectable levels of the virus in circulation. Thus, further characterization of antiretroviral nanoparticle biodistribution is warranted.

Defining delivery for HIV-1 therapeutic agents to lymphoid tissues could aid in the design of more effective clinical outcomes. To this end, theranostics serve as a bridge between therapeutic and diagnostic tracking. Previous iterations of HIV-1 theranostics included spherical europium cobalt-ferrite (CFEu) 14 and bismuth sulfide nanorods (BSNRs) 15. These particles, loaded with RPV 14, 15 or dolutegravir (DTG; an INSTI) 16, showed characteristics of enhanced particle uptake in macrophages. Real-time non-invasive tracking of nanoparticles biodistribution using fluorescence, magnetic resonance imaging (MRI), and single photon emission computed tomography (SPECT-CT) conferred by their metal cores or inserting radiolabel tags have allowed the prediction of drug biodistribution based on early bioimaging composite analyses 15.

Taken together, theranostics provide a platform to construct nanoparticles in pursuit of sustained HIV suppression, a prerequisite for viral elimination from organisms 17. Herein, we tested the concept that theranostics and antiretroviral nanoparticles with similar sizes and shapes will display parallel biodistributions. A novel theranostic agent, europium sulfide (EuS), was deployed as a cellular uptake model. EuS nanoprobes elucidate a potential dynamin-mediated mechanism for direct nanoparticle endocytosis by CD4+ T cells, a principal target of HIV-1. Real-time and non-invasive molecular imaging also reveals the utility of EuS as companion particles for NRPV in trafficking nanoparticles to known reservoir tissues, such as the spleen. These advances signal how nanotheranostic agents may serve as surrogates to better design long-acting ART and clustered regularly interspaced short palindromic repeats (CRISPR) nanoformulations for therapeutic delivery and viral elimination.

## Methods

### Materials

RPV was purchased from LeapChem (Hangzhou, China). RPV nanoformulations (NRPV) were manufactured using an Avestin EmulsiFlex-C3 high-pressure homogenizer (Ottawa, ON, Canada). NRPV was prepared to replicate RPV LA developed by Janssen 18, as described previously 19. UPLC-MS/MS parameters for drug concentration determinations were performed as reported 19 and are detailed in supplementary materials. L-α-phosphatidylcholine, Tween-80, europium (III) chloride hexahydrate, 1-dodecanethiol, oleic acid, and oleylamine were obtained from Sigma-Aldrich. ^177^LuCl_3_ was obtained from the National Isotope Development Center, Oak Ridge National Laboratory, Oak Ridge, TN, USA. DSPE-PEG_2000_ was purchased from Corden Pharma.

### Cells

CEM-ss CD4+ T cells (NIH AIDS Reagent Program #776) were cultured in RPMI 1640 containing 2 mM L-Glutamine, 10% fetal bovine serum, and 100 U/mL penicillin plus streptomycin (PenStrep). Monocytes were obtained from human HIV-1/2 and hepatitis seronegative donor elutriation without person identifiers and was thus exempt from the University of Nebraska Medical Center (UNMC) Institutional Review Board review. Cells were differentiated into monocyte-derived macrophages (MDM) by culture in Dulbecco's Modified Eagle Media (DMEM) containing 4.5 g/L glucose, 10% pooled human serum, 1000 U/mL macrophage colony-stimulating factor, 1% glutamine, 50 μg/mL gentamicin, and 10 μg/mL ciprofloxacin for 7 days.

### Animals

Male Balb/c mice (8 weeks old) were purchased from Jackson Laboratories (Bar Harbor, Maine, USA). Animals were housed in the UNMC Comparative Medicine animal facility according to the Association for Assessment and Accreditation of Laboratory Animal Care guidance. The UNMC Institutional Animal Care and Use Committee approved all protocols related to animal experiments and were certified to have met the requirements and ethical guidelines set forth by the National Institutes of Health in handling laboratory animals for research.

### Synthesis of ^177^LuEuS particles

Intrinsically radiolabeled ^177^LuEuS particles, were prepared by a solvothermal technique in an isolated radioactive workstation. Europium (III) chloride hexahydrate (0.2 mM, ~72 mg), oleic acid (0.5 ml), and oleylamine (4 ml) were mixed in a glass vial and this mixture was stirred for 30 min, and completely dispersed by bath sonication. Then ~70 mCi of ^177^LuCl_3_ and 1-octadecene (5 mL) were mixed in a separate vial and transferred to the europium-oleic acid-oleylamine (Eu-OA-OAm) mixture. 1-Dodecanethiol (3.0 mL) was quickly added to the reaction medium. The mixture was sonicated for 15 minutes to provide a completely homogenous mixture. After vigorous stirring for 10 min, the solution was transferred into a high safety Teflon-lined reactor placed in a stainless-steel autoclave reactor, sealed airtight, and heated at 290°C for 1 hour. The systems were then allowed to cool naturally to room temperature (around 8 hours). After reaching room temperature, the reactor was opened in a controlled radioisotope workstation. Fifty milliliters of ethanol were added, vortexed and sonicated. The solution was then centrifuged at 976 x g for 30 minutes at 15°C. ^177^LuEuS particles were collected and purified by repeating the same ethanol washing process three times to remove non-reacted components. The radioactive concentration of gamma-ray emitting lutetium-177 in stock ^177^LuEuS was measured using a gamma counter (CRC^®^-25R dose calibrator, Capintec, Inc, Florham Park, NJ, USA) (activity was ~ 60 mCi). Lipid coated ^177^LuEuS particles were prepared by thin-film dispersion technique. First, 100 mg L-α-phosphatidylcholine and 50 mg DSPE-PEG2000 were dissolved in a chloroform/hexane mixture (5:1 v/v) contained within a round-bottomed flask and evaporated to form a thin film using a rotavap (Heidolph, Hei-VAP Precision; G3B Vertical, USA). The films were dried under vacuum overnight to complete the removal of organic solvents. Second, ^177^LuEuS particles were dispersed in chloroform (4 mL) followed by bath sonication for ~ 10 min to get a uniform dispersion of the particles in the solvent. The ^177^LuEuS- chloroform dispersion was mixed with 3 mL of 2% (v/v) Tween-80 in a 25 mL round-bottom flask and sonicated for 5 minutes to form a turbid metalosome emulsion. Third, chloroform was removed from the ^177^LuEuS/chloroform/Tween-80 emulsion by rotaevaporation. Lastly, the ^177^LuEuS particle solution was transferred to the lipid film-coated round-bottomed flask and dispersed in the lipid film through gentle rotation at 35°C with continuous bath sonication. Lipid coated ^177^LuEuS was used for radioactivity analyses. Non-radiolabeled EuS particles were synthesized by the same procedure lacking ^177^LuCl_3_ as a reactant.

### Radiolabeled particle stability

The *in vitro* stability of lipid coated ^177^LuEuS particles was determined by spiking 10.2 μCi (~ 100 μL) of ^177^LuEuS in tubes containing 400 μL Balb/c mouse serum incubated at 25°C or 37ºC for seven days. The samples were centrifuged at 77,474 × g for 30 minutes at 10ºC. The supernatant was collected into a separate microcentrifuge tube. The radioactivity in the pellet and the supernatant was measured by gamma counter. To determine percent stability the following equation was used: Radiolabeling stability (%) = radioactivity in pellet/total radioactivity (pellet plus supernatant) × 100.

### Cellular uptake of NRPV and EuS

For CD4+ T cell studies, individual wells of 12-well tissue culture plates were precoated with 100 μg/mL poly-L-lysine for one hour and then the contents were removed and washed once with sterile PBS. CEM-ss CD4+ T cells or MDM were seeded in 12-well plates at 1 × 10^6^ cells / well in 1 mL media. The non-cytotoxic concentration of EuS was determined by absorbance at 490 nm after cells were treated with serially diluted EuS for 24 hours, followed by 2-hour incubation with 3-(4,5-dimethylthiazol-2-yl)-2,5-diphenyltetrazolium bromide (MTT, 1 mg/mL). T cells and MDM received lipid-coated EuS particle treatments at 10 ppm and 100 ppm of Eu-153 (Eu^153^), respectively. Chemical inhibitors were spiked into appropriate wells at final concentrations of 30 μM Pitstop2, 100 μM Dynasore, or 4 μM CK666. At designated time points, supernatants were discarded, and cells were washed 3× with 1 mL PBS and counted by trypan blue exclusion staining. After that, collected contents were centrifuged 650 × g for 5 minutes at 4°C to pellet nanoparticle-containing cells. Pellets were stored for quantitation of inorganic material (ICP-MS) or drug (UPLC-UV/Vis), as detailed in supplemental materials.

### ^177^LuEuS biodistribution

SPECT-CT imaging was performed on mice to assess the *in vivo* biodistribution of ^177^LuEuS particles. Each animal was dosed by tail-vein injection with ~ 500 μCi of ^177^LuEuS (volume of injection: 170 μL) plus 45 mg/kg NRPV (volume of injection: 100 μL). Images were acquired at 6, 12, 24, 48, and 120 hours post injection using a SPECT-CT system (Flex Triumph, TriFoil Imaging, Northridge, CA, USA) fitted with a five pinhole (1.0 mm per pinhole) collimator. CT images were acquired using 360 projections over 360° with an X-ray tube current of 140 mA and voltage of 75 kilovoltage peak (kVp) at a magnification of 2.0 (field of view = 59.2 mm^2^). Immediately after, SPECT image acquisition was performed with the following parameters: 64 projections at 15 seconds per projection over 360° using a radius of rotation of 48 mm (field of view = 59 mm^2^). Co-registration of anatomical CT images and functional SPECT was performed by 3D visualization and analysis software, VivoQuant 3.5 (Invicro Boston, MA, USA). Regions of interest (ROIs) were drawn over various organs and radioactivity content and organ volumes were determined and used to calculate counts per cubic millimeter (mm^3^). Mice were sacrificed 5 days post-injection. Heart, lungs, liver, pancreas, stomach, spleen, small intestine, large intestine, kidneys, bladder, lymph nodes, muscle, bone, brain, testes, injection site (tail) and blood were collected, weighed, and analyzed for radioactivity using gamma scintillation spectrometry (ORTEC NaI(Ti) Scintillation Radiation Detector, Ametek, Oak Ridge, TN, USA). Additionally, animals were individually caged so that excreted signal could be measured from the bedding. Signal measured was back calculated to the injection time to account for the radioactive decay of ^177^Lu (t_1/2_ = 6.64 days). The signal from each tissue was normalized to the sum of total counts from all sources and then divided by the weight of the organ to obtain % injected dose (ID)/g for each tissue.

### MRI relaxivity measures

EuS particle phantoms for MRI measures were prepared as previously reported 20. MRI data were acquired on a 7T/16 cm Bruker PharmaScan MRI system (Bruker; Ettlingen, Germany). For T2 map measurements, CPMG (Carr Purcell Meiboom Gill sequence) phase-cycled 3-dimensional multi-echo sequence data was acquired with 250 ms repetition time, 48 echoes (echo times TEn = n X 2.618; n = 1,…,48), 128 × 128 × 64 acquisition matrix, 70 × 64.76 × 42.38 mm FOV, one average, for a total scan time of 34 min.

### Animal MRI

Animal MRI was performed on a 7T/16 cm Bruker PharmaScan MRI system. Mice were anesthetized with isoflurane in 100% oxygen and maintained at 40-80 breaths per minute. Animals were scanned by MRI prior to or after dosing with EuS (17 mg/kg, tail vein injection). T2-relaxtion maps were generated using a CPMG phase-cycled multi-echo multi-slice sequence. Data were acquired with 3000 ms repetition time, 50 echoes (echo times TEn = n × 10 ms; n = 1), 256 × 128 acquisition matrix, 25 × 25 mm FOV, slice thickness = 0.5 mm, 2 averages, for a total scan time of 13 min. T2 signal in liver, spleen, and kidneys was quantified by densitometry of ROI pixilation in ImageJ v1.51m9 (National Institutes of Health, USA) 21 and converted to ΔR2 as 1000/(T2_post-treatment_ - T2_pre-treatment_).

### Statistical analyses

Linear regressions and statistics were calculated in GraphPad Prism v7.0 (La Jolla, CA) as described in the figure legends. Data are depicted as mean ± standard error of the mean (SEM) of biological triplicates. Statistical significance was measure by two-way ANOVA with Bonferroni corrections for multiple comparisons.

## Results

### Synthesis and characterization of radiolabeled EuS (^177^LuEuS) nanoparticles

**Figure 1:**
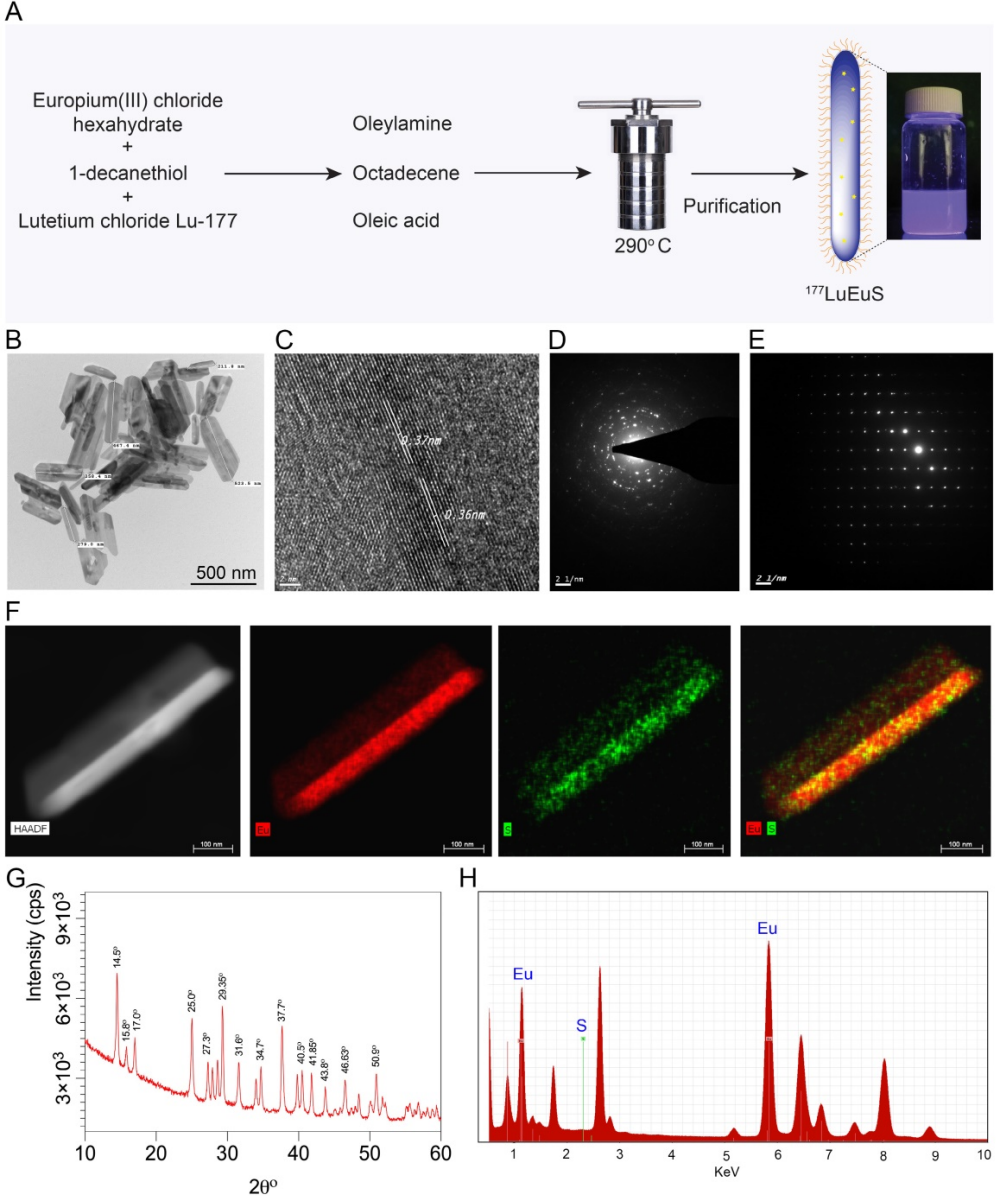
**Synthesis and characterization of EuS particles.** (A) Synthesis scheme of EuS particles. 1-Dodecanethiol served as a reducing agent plus sulfur precursor; the vial shows particle fluorescence. (B) Low-resolution TEM image of EuS is illustrated. The EuS dimensions were approximately 25 nm in diameter by ~ 350-500 nm in length. (C) HR-TEM images of a single EuS particle with lattice planes of 0.37 nm, corresponding with XRD data. (D-E) The selected area electron diffraction (SAED) pattern of EuS show characteristic interplanar spacing of the EuS single crystal structure. (F) The europium (red) and sulfur (green) element STEM mapping showed element localization within the particles by corresponding high-angle annular dark-field electron microscopy. (G) XRD patterns, correlating with HR-TEM lattice pattern and SAED data sets, confirmed the particles' structural configurations. (H) Chemical composition of EuS particles assessed by EDX qualitatively demonstrated the particle elements.

Intrinsic “chelator-free” and non-leachable radiolabeled europium sulfide (^177^LuEuS) was first synthesized by solvothermal precipitation of europium (III) chloride hexahydrate and 1-decanethiol in the presence of oleic acid, oleylamine and octadecene with ^177^LuCl_3_ at 290°C **(Figure 1A)**. Parallel reactions were performed without ^177^LuCl_3_ to enable non-radioactive experiments. Lipid coatings containing L-α-phosphatidylcholine and DSPE-PEG_2000_ were used to make biocompatible lipid coatings for EuS particles. Transmission electron microscopy (TEM) revealed that EuS nanocrystals had rod shapes of 25 nm in diameter by ~ 350 nm in length (**Figure 1B**). These approximate the 250 nm and 350 nm hydrodynamic diameters of NRPV, and RPV prodrug formulations (NM3RPV) as described previously 19. High resolution (HR)-TEM images **(Figures 1C-E)** show the lattice fringes with spacings of 0.36-0.37 nm. High-angle annular dark-field scanning TEM (HAADF-STEM) illustrates europium (Eu; red) and sulfur (S; green) as an approximate 70%:30% composition (**Figure 1F**).

The X-ray diffraction (XRD) shown in **Figure 1G** reveals that all peaks are indexed into a cubic crystal and reconfirmed with the JCPDS file for Eu and S or bulk EuS (JCPDS file numbers; 01-073-6349;26-1419; 05-001-0200; 04-007-2069). The XRD peaks of 14.5°, 15.0°, 17.0°, 25.0°, 27.3°, 29.35°, 31.6°, 34.7°, 40.5°, 41.85°, 43.8°, 46.63° and 50.9° at 2θ are typical indications for face-centered cubic planes of EuS crystal structure. For example, space group (111 = hkl) lattice spacing approximate 3.6 Å = 0.36 nm, corresponding to 25.0° in 2θ and 0.36 nm spacing obtained through HR-TEM (**Figures 1C-E**). The energy dispersive X-ray spectroscopy (EDX) analysis demonstrated “europium” and “sulfur” signals were detected in the EuS nanoprobes (**Figure 1H**). Results cumulatively illustrate successful synthesis of rod-shaped nanoprobes whose elemental core constituents are readily detectable after formulation.

### Multimodal trackability of EuS Nanoparticles

**Figure 2:**
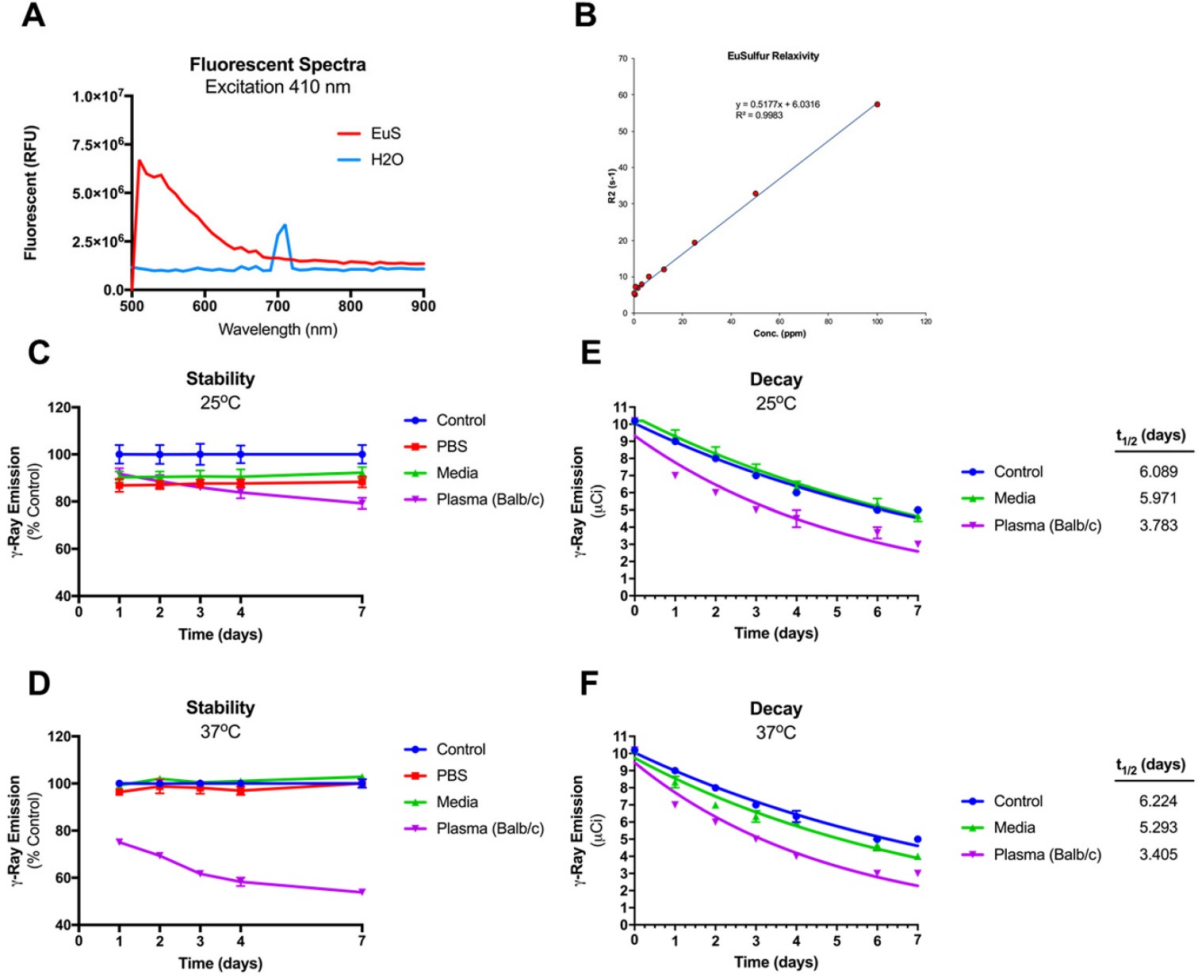
** Intrinsic tracking properties of europium sulfide (EuS) nanoprobes.** (A) Fluorescence spectra of resuspended EuS at λ_max_ 410 nm excitation. (B) R2 relaxation rate (inverse T2 value) was determined using serial dilution of EuS phantom particles in agar matrix. (C-F) Integrity of ^177^Lu intrinsic doping in EuS nanoparticles was determined by dosimetry. (C-D) Stability was calculated by normalizing radioactivity of experimental pellets against those of control. (E-F) The half-lives of ^177^LuEuS were calculated using one-phase decay algorithms of radiation levels across times using GraphPad Prism v7.0*.*

To ascertain the potential of EuS as surrogates for NRPV tracking *in vivo*, the nanoprobes were tested for compatibility with a range of bioimaging modalities. Fluorescence provides a sensitive means of detection across short distances. Fluorescent blue spectra for EuS revealed maximal excitation and emission wavelengths at 410 nm and 510 nm, respectively (**Figure 2A**). EuS fluorescence is due to electron transitions from the 4f7 ground state to 4f6 5d1 excited state (Eu(II)) 22. Similarly observed results from europium-containing nanotubes have been attributed to a +2-oxidation state that yields blue light upon UV wavelength excitation 23.

Intact NRPV likewise emits fluorescence, albeit at a shorter wavelength (385 nm), resulting from aggregation induced emission 24. The MRI compatibility of EuS was also determined as a means of quantifying particle accumulation at different tissue depths. At issue lies the propensity of f → d transition block electrons to preferentially oxidize Eu^2+^ to Eu^3+^, whose MRI relaxivity is short-lived 25. The T2-weighted relaxivity constant of EuS nanoprobes measured 0.5177 ppm/second and showed strong linear correlation with europium concentration (**Figure 2B**). High T2-weighted relaxivity paired with blue wavelength emission suggests coordinated electron stability conferred to europium by sulfide atoms. Finally, gamma-ray emitting ^177^Lu was intrinsically doped into the cores of EuS nanoprobes to provide a final means of sensitive biotracking via SPECT-CT. The particle stability and half-life of ^177^LuEuS was found to be ~3.4 days under physiological conditions (37^o^C in serum matrix), which represents a 45-50% reduction from the half-lives of control-incubated sample (6.2 days) and native ^177^Lu (6.7 days) (**Figures 2C-F**). The radioactivity profile of ^177^LuEuS indicates that the nanoprobes are compatible with SPECT-CT imaging over a short duration.

### Endocytic Pathways of EuS Nanoparticle Uptake

**Figure 3:**
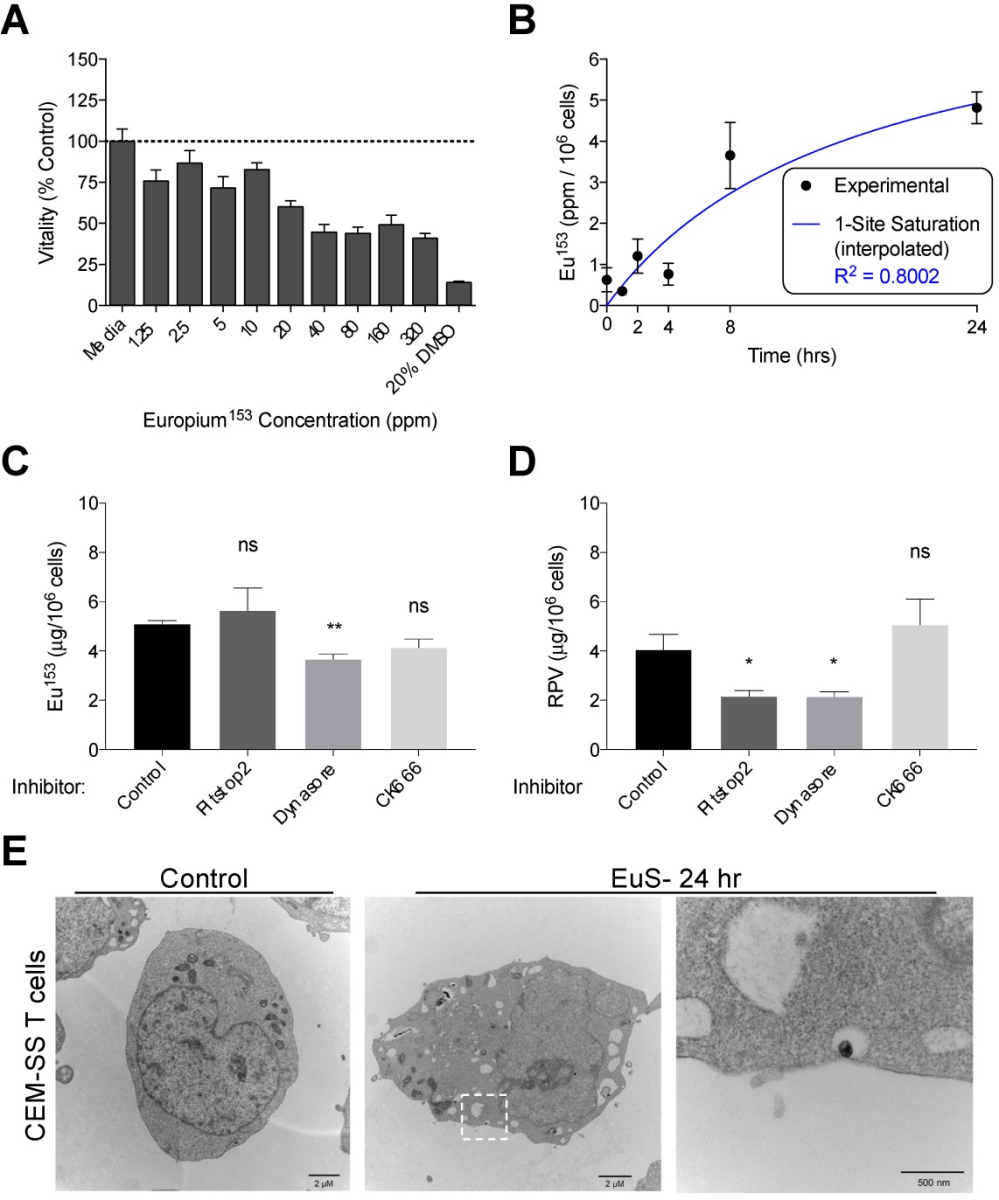
**Dynamin-mediated endocytosis of EuS nanoparticles by CEM-ss T cells.** (A) EuS cytotoxity in CEM-ss T cells after 24-hour incubation was determined by MTT assay. (B) Time course of EuS uptake when treated at 10 ppm Eu^153^ was measured by ICP-MS. Saturation kinetic curve was fitted to experimental data using 1-site saturation curve in GraphPad Prism v7.0. (C-D) The mechanisms of NRPV and EuS nanoparticle uptake were assessed by pre-treating with clathrin-inhibitor (Pitstop2, 30 µM), dynamin/caveolin-inhibitor (Dynasore, 100 µM), or actin-mediated active transport inhibitor (CK666, 4 µM). (E) Transmission electron micrographs of CEM-ss T cells in the absence (left) or presence (center) of EuS. Far right micrograph (inset of center) depicts nanoparticle within endocytic pit. Bar graphs depict mean ± SEM of three biological replicates. Statistical differences were determined using two-way ANOVA between groups with a Bonferroni's post hoc-test to correct for multiple comparisons. *p < 0.05; ns: non-significant.

We next examined the propensity for HIV-1 infectible CD4+ T cells and monocyte-derived macrophages (MDM) to endocytose EuS nanoparticles. A non-cytotoxic concentration of 10 ppm Eu^153^ was determined by mitochondrial vitality assay over 24 hours in CEM-ss CD4+ T cells (**Figure 3A**). A time course of EuS nanoparticle uptake in CEM-ss cells revealed 52% uptake of 10 ppm Eu^153^ treatment by 24 hours (**Figure 3B**). The one-site saturation kinetic modeling of EuS uptake indicates delayed nanoparticle entry that becomes apparent between 4 and 8 hours post-treatment. To investigate a possible mechanism for EuS nanoprobe endocytosis by CD4+ T cells, a 24-hour uptake experiment was repeated in the presence of chemical inhibitors. Clathrin, dynamin, or actin related protein 2/3 (Arp 2/3) complexes were blocked with Pitstop2 26, Dynasore 27, or CK666 28, respectively. Dynamin blockade with Dynasore elicited reductions in NRPV (50%) and EuS (35%) uptake (**Figure 3C-D**).

Notably, clathrin inhibition by Pitstop2 also attenuated NRPV accumulation in CD4+ T cells (**Figure 3C**). The reduction of EuS uptake with dynamin inhibition (**Figure 3D**) paired with the appearance of endocytic pits by TEM (**Figure 3E**) suggests that caveolin rather than clathrin or actin line the cytoplasmic surface of CD4+ lymphocyte cellular invaginations 29.

**Figure 4:**
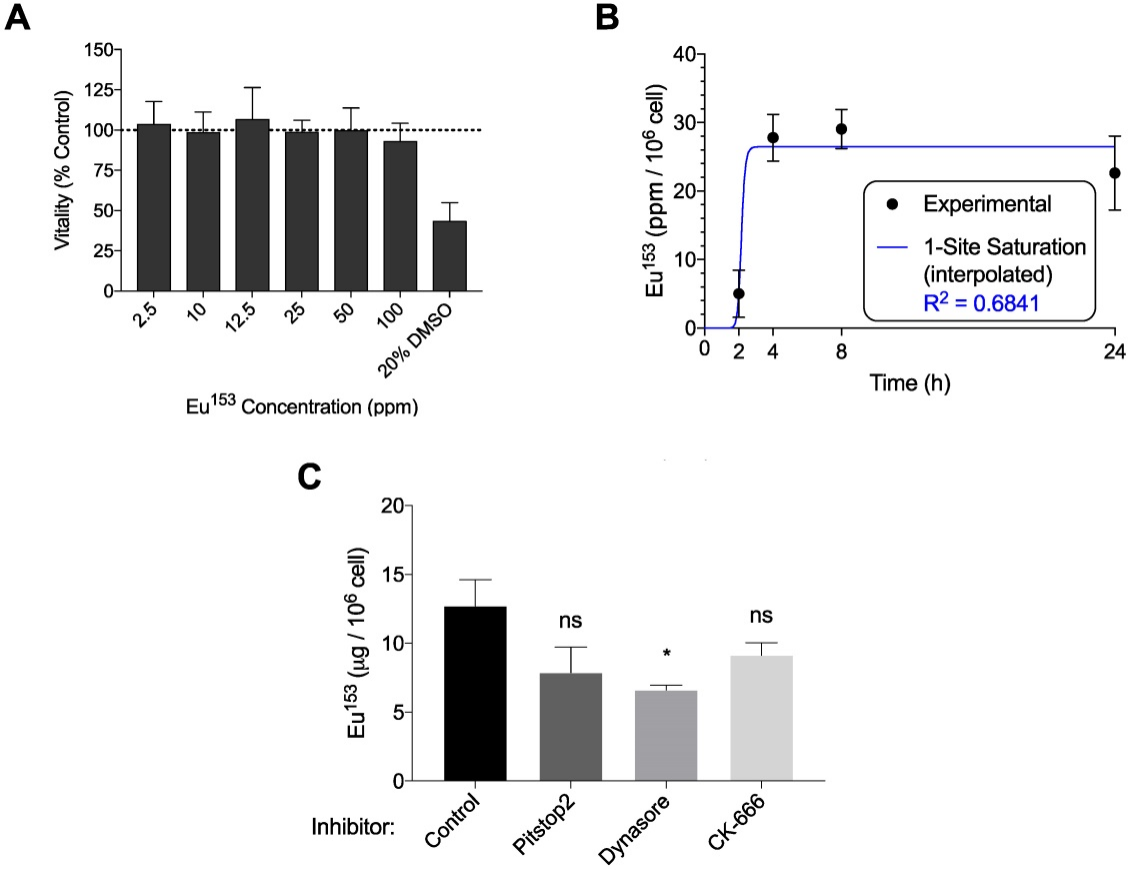
** EuS uptake in human monocyte-derived macrophages (MDM).** (A) EuS cytotoxicity in MDM after 24-hour incubation was determined by MTT assay. (B) Time course of EuS uptake of cells treated with 100 ppm Eu^153^ over 24 hours as measured by UPLC-TUV. The saturation kinetic curve was fitted to experimental data using 1-site saturation curve in GraphPad Prism v7.0. (C) The mechanism of nanoparticle uptake was assessed by pre-treating with Pitstop (30 µM), Dynasore (100 µM), or CK666 (4 µM) endocytic inhibitors. Graphs depict mean ± standard error of the mean (SEM) of three biological replicates. Statistical differences were determined using two-way ANOVA between groups with a Bonferroni's post hoc-test to correct for multiple comparisons. *p < 0.05; ns: non-significant.

Macrophages, which serve as long-term depots for nanoformulated antiretroviral prodrugs, were likewise assayed for phagocytosis of EuS. MTT assessment indicated no decline in mitochondrial vitality at concentrations up to 100 ppm Eu^153^ (**Figure 4A**). MDM displayed maximal uptake between 2-4 hours post treatment, with 28% of the added EuS particles taken up by the cells by 4 hours (**Figure 4B**). These levels approximate the 10% NRPV uptake reported in MDM previously 24. Co-treatment with Dynasore significantly reduced EuS uptake in MDM by 40% (**Figure 4C**). Together, these data point to enhanced and rapid uptake of EuS by macrophages in a caveolin-dependent manner.

### EuS nanoparticle real time biodistribution

**Figure 5:**
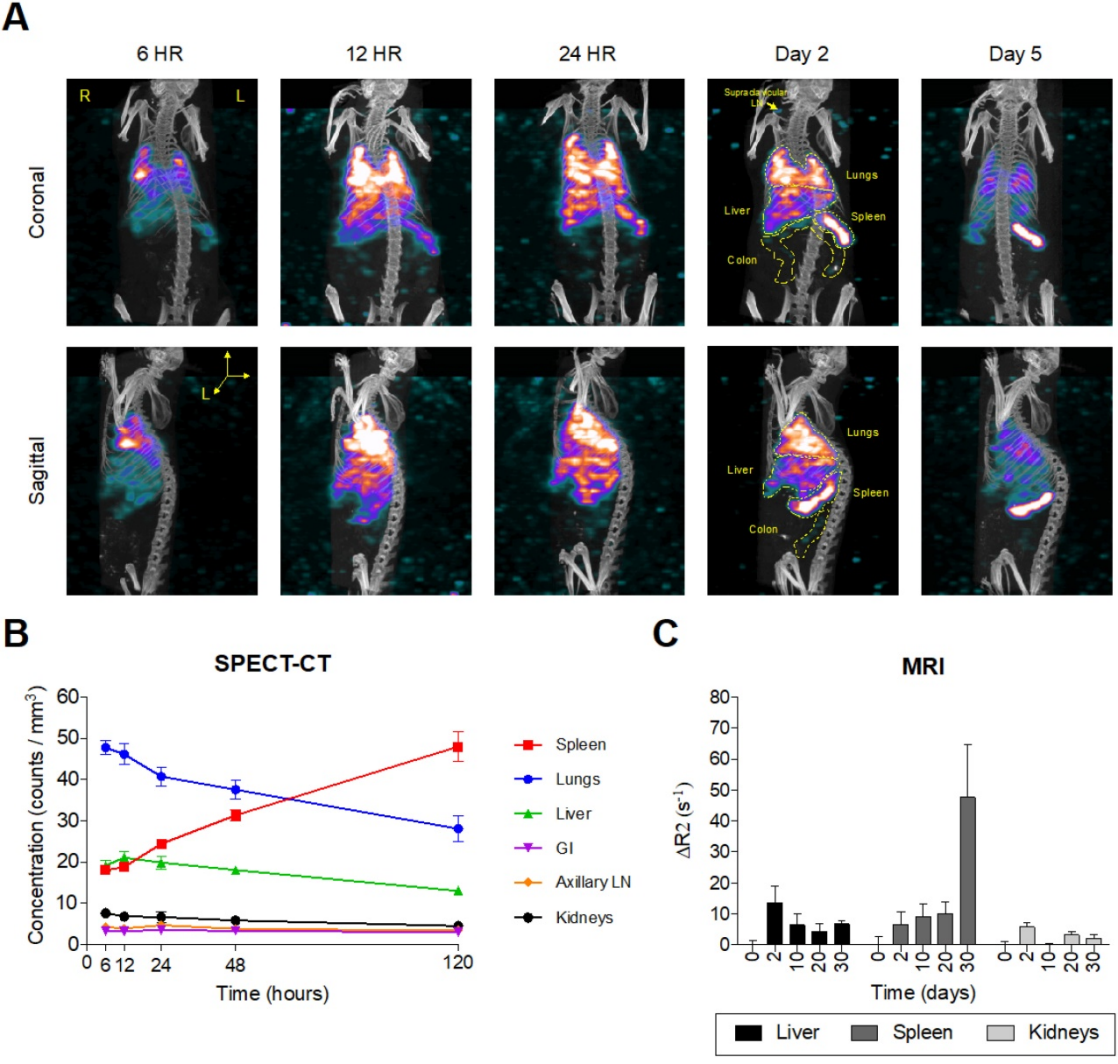
** Europium-sulfide (EuS) nanoparticles redistribute to spleen.** Male Balb/c mice (n = 5) were tail vein injected with ~ 500 µCi ^177^LuEuS nanoparticles (17 mg/kg europium-153) or EuS (17 mg/kg europium-153) plus NRPV (45 mg/kg). (A) SPECT-CT images and (B) signal concentrations in ROIs were processed using VivoQuant v3.5 (Invicro Imaging Software) based on anatomy observed in plain CT. (C) Differences in R2 relaxivity after dosing (ΔR2) based on densitometry in ROIs drawn in ImageJ were calculated. Plots represent averages ± SEM.

Intrinsically doped ^177^LuEuS particles were assessed for their acute biodistribution upon intravenous (IV; via tail-vein) injection. To accelerate redistribution from muscle depots formed by long-acting antiretroviral intramuscular administration, mice were dosed sequentially with NRPV (45 mg/kg) followed by EuS nanorods (~500 μCi ^177^LuEuS/mouse or 17 mg Eu^153^/kg) via tail-vein injection. Bioimaging by SPECT-CT revealed an initial accumulation of nanoprobes in lungs by 24 hours (**Figure 5A-B**). This finding was unexpected but indicative that the 300-500 nm length of our particles likely caused them to become entrapped in pulmonary capillaries during IV administration. By 48 hours, ^177^LuEuS were readily imaged in known HIV-1 anatomic reservoirs including spleen, liver, lungs, colon, and lymph nodes. SPECT-CT performed 5 days post-dosing illustrated the highest accumulation of ^177^LuEuS in spleen.

An extended 30-day time course was used to examine EuS nanoprobe biodistribution via MRI. T2-weighted images revealed initial accumulation of EuS in liver two days post dosing, with significant redistribution to spleen between days 10 and 30 (**Figure 5C**). The limitations of T2 contrast nanoprobes was observed by the lack of enhancement in lungs, ileum, colon, lymph nodes and brain. These results highlight the value in designing rod-shaped nanoparticles for trafficking payloads to the spleen, a known secondary lymphoid organ.

### EuS accumulation in organs approximates NRPV biodistribution

**Figure 6:**
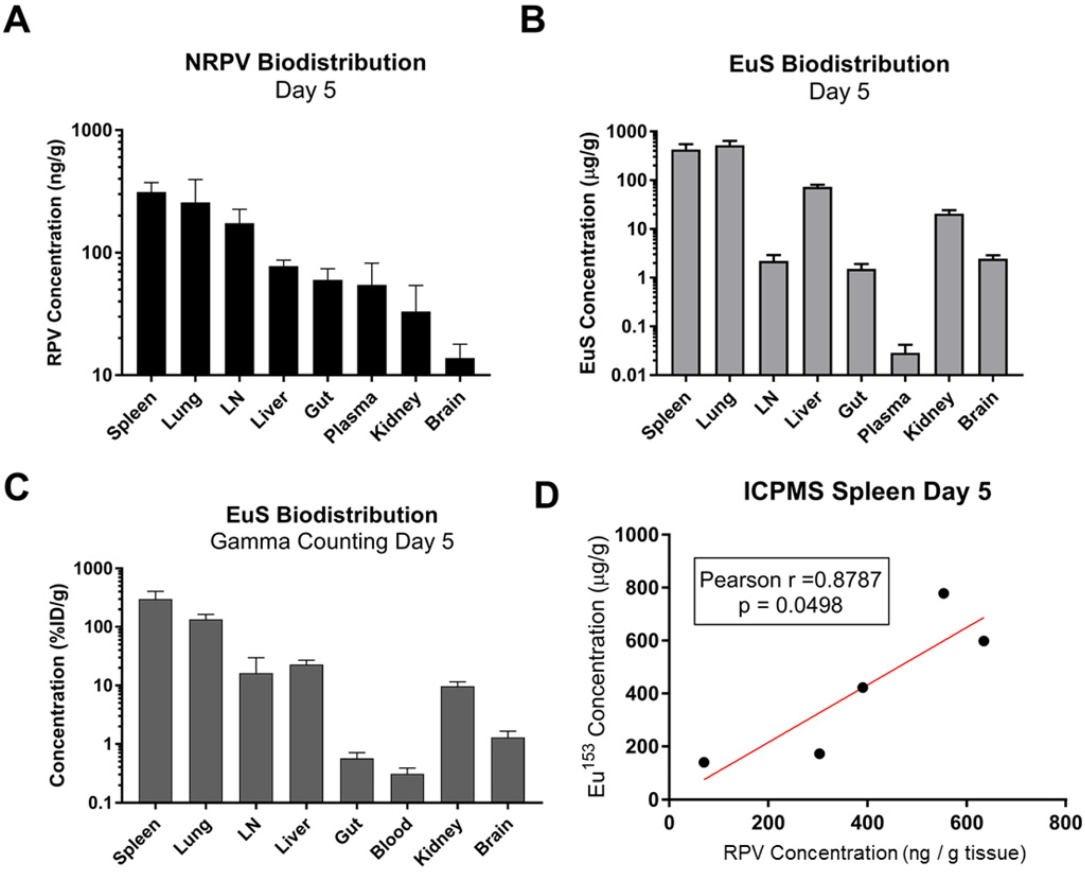
** Quantitative analysis of RPV and EuS in mouse tissues 5 days post-dosing.** (A) NRPV drug concentration was measured in various tissues by UPLC-MS. (B) Europium-153 concentration was quantitated by ICP-MS. (C) Gamma-ray emission from collected tissues was measured after briefing radioactive decay by scintillation counting. (C) Raw radioactivity was divided by total radioactivity in all tissue measured per animal, giving percent injected dose, which was further normalized against tissue weight (% ID/g). (D) Pearson correlation between NRPV drug concentration values and europium-153 was determined in day 5 spleen samples.

Comparative analyses were performed to determine the utility of EuS nanoprobes as predictive biotrackers for NRPV distribution. Organs collected at sacrifice 5 days post-dosing revealed greatest accumulation of RPV in spleen (313 ng/g tissue), lung (257 ng/g tissue), lymph nodes (174 ng/g tissue), and liver (78 ng/g tissue) (**Figure 6A**).

Europium-153 concentrations were also observed to be highest in the spleen (428 μg/g tissue), lung (525 μg/g tissue), and liver (73 μg/g tissue) by day 5 as measured by ICP-MS (**Figure 6B**). Parallel findings were observed using gamma-ray scintillation counting, which reflects ^177^Lu content (**Figure 6C**). Lower metal contents observed in lymph nodes at day 5, as compared to RPV, could result from variability in the sizes and locations from which lymph nodes were collected at the time of harvest.

**Figure 7:**
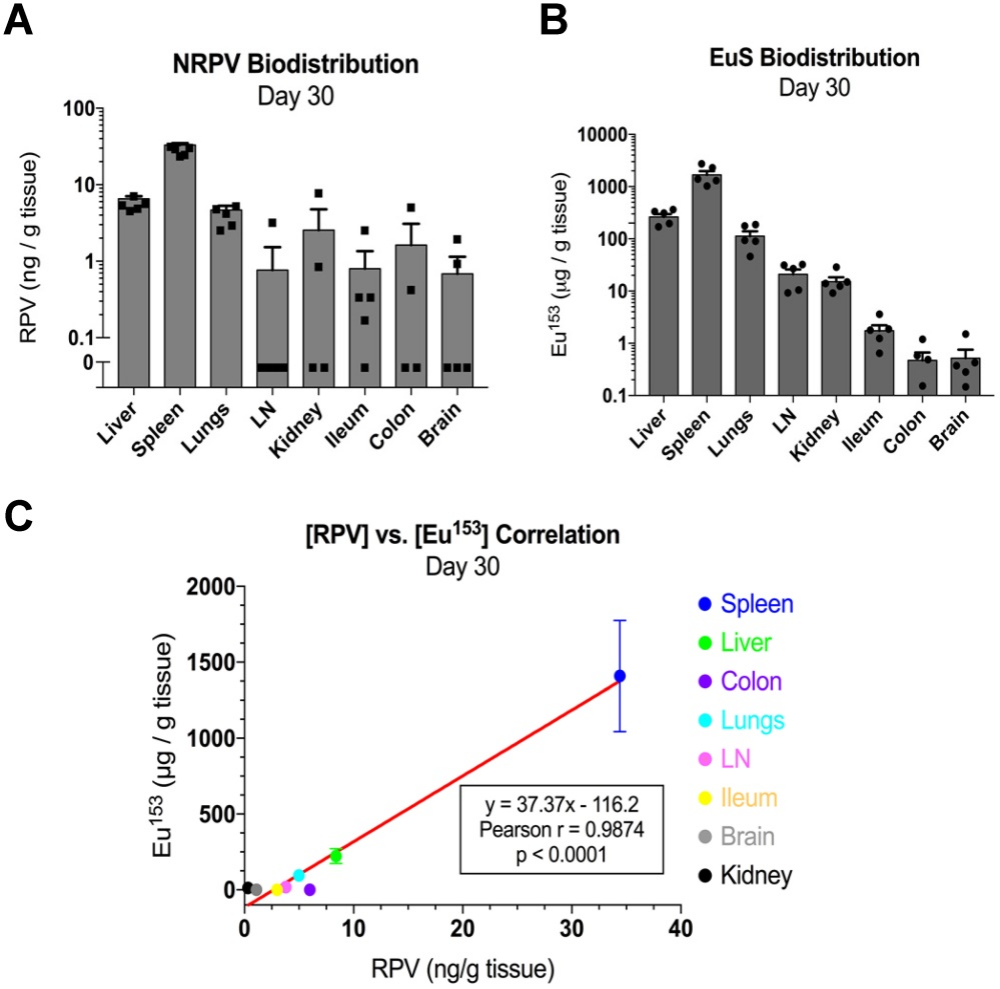
** EuS correlates with NRPV biodistribution 30 days post-dosing.** (A) NRPV drug concentration was measured in various tissues by UPLC-MS/MS. (B) Europium-153 concentration was quantitated by ICP-MS. (C) Day-30 Pearson correlation was calculated between average RPV drug concentration and europium-153 concentration for each organ.

Mass spectrometry quantitation for drug and metals performed at day 30 post-dosing revealed similar accumulations to those found at day 5. In particular, RPV was readily detectable in spleen (33 ng/g tissue), liver (9.3 ng/g tissue), and lungs (5 ng/g tissue) (**Figure 7A**). Europium-153 measured 1409 μg/g tissue in spleen, 223 μg/g tissue in liver, and 95 μg/g tissue in lungs (**Figure 7B**). Replicate values for NRPV and EuS in colon and brain approached assay detection limits. The comparable distribution of both particles to macrophage rich organs suggests phagocytic processes are vital in determining the biodistribution of theranostics.

Pearson correlations validated the significance of these trends at early and late timepoints. Spleens from dual-treated mice demonstrated a correlation between RPV and europium-153 concentrations (p = 0.0498; **Figure 6D**). This may relate to differences in organ perfusion or overall animal health in the context of dosing with radioactive materials. Meta-analysis from terminal sacrifice also displayed a significant correlation (p < 0.0001) between rilpivirine and europium-153 present in each organ (**Figure 7C**). As the high accumulation of both EuS and NRPV in spleen anchored this trend, it is possible that the utility of EuS in predicting drug content deposits in organs that serve as minor depots for ART is limited.

## Discussion

Long-acting antiretroviral nanoformulations serve to improve therapeutic outcomes for treatment of HIV-1 infection and for prevention through PrEP. We posit that the development of such therapeutics can be accelerated through nanotheranostics. Here, defining delivery of drugs to cell and tissue reservoirs could lead to improved viral suppression, reduced transmission and CRISPR-associated HIV-1 elimination. As a first step to achieve these goals we investigated the delivery of the newly United States Food and Drug Administration approved long acting antiretrovirals. This includes RPV, which with CAB, has demonstrated non-inferiority as part of HIV-1 maintenance therapy 3. Given that long-acting ART injections are not indicated for induction therapy, we sought to characterize RPV nanoparticles' ability to reach viral reservoir target sites. Sensitive nanoprobes with bioimaging capabilities offer a surrogate means to trace antiretroviral biodistribution while reducing the cost, labor, and time otherwise required to survey drug concentrations at tissues of interest. Herein, we validated the utility of EuS nanoparticles as mimetics for NRPV cellular uptake and biodistribution.

Although macrophages readily phagocytose antiretroviral nanoparticles, ART efficacy is in part a property of cellular uptake. Prior works revealed that rod-shape, use of poloxamer excipients, and surface decoration with folate, all modestly enhance ART nanoparticle uptake by macrophages 30, 31. Theranostics have recapitulated these findings 16, 20. Here we present dynamin/caveolin as a primary endocytic pathway for nanoparticle entry into MP, distinct from previously reported clathrin-mediated endosomal trafficking of protease-inhibitor nanoparticles 32, 33. Nanoformulated drugs accumulate in late endosomes where HIV-1 virions are present in high abundance 33, 34. Antiretroviral prodrug nanoformulations are also present in high abundance within late- and recycling endosomes 35, 36. We postulate that while nanoformulated antiretrovirals and long-acting prodrugs both meet their targets in endosomes, prodrugs are hydrolyzed to their active forms under increasingly acidic late endosomal and lysosomal microenvironments 37 whereas native ART become degraded. HIV-1 and antiretroviral drug nanoparticles employ recycling endosomes for sustained release from cells 38. Accordingly, all therapeutic nanoparticles for HIV-1 treatment should be engineered to withstand acidification in late endosomes.

An alternative to macrophage-mediated delivery rests in the direct uptake of particles by CD4+ T cells. As dynamin but not clathrin blockade significantly impeded EuS and NRPV uptake, we conclude that CD4+ T cells endocytose nanocrystals in caveolin-coated pits which operate in tandem with dynamin “pinching” of invaginated surfaces during endocytic vesicle formation. This finding accords with other studies that describe dynamin-2 as critical in CD4+ T lymphocyte endocytosis of T cell receptors 39 and HIV-1 40. Dynamin-2 enables HIV-1 transmission between adjacent T cells 40 and triggers appropriate clonal expansion via mTOR-cMyc signal cascades when TCR encounters cognate antigen 39. As such, dynamin likely serves as an important cytoskeletal protein involved in transmission of vesicle-bound materials within its limited cytoplasm. Inclusion of rare earth metals, like europium, in therapeutic nanoparticles would confer the ability to track its migration through T cells' endosomal network via fluorescence.

Theranostic drug particles play an essential role in tracking therapeutic cargo *in vivo*. The rod shape and size of EuS matches the morphology of NRPV but maintains inherent fluorescent and magnetic properties. EuS was modified with lipids and transformed from lipophobic to lipophilic to expand tracking. The simplified EuS design, which eliminates the need for transition metals to complex with europium, conferred predictive multimodal trackability over a month. Unlike previous nanoprobes bearing cobalt-ferrite cores 14, 16, sulfur atoms sufficiently stabilize the europium (II) oxidation state. This enhanced its blue-wavelength fluorescence and T2-weighted contrast capabilities. Sulfur is a preferred coordinate binding partner to other inorganic elements as a result of its high biocompatibility 41, 42, antioxidant capacity 43, and low cost. Europium-153 concentrations correlated with RPV content in macrophage rich organs including spleen, liver, and lungs. Accordingly, EuS proved useful as a surrogate for RPV concentration for up to 30 days, particularly in splenic tissues. Non-invasive SPECT-CT and MRI revealed nanoprobe redistribution from lung to macrophage-rich tissues including liver and spleen. Our prior studies showed detectable RPV levels in liver, spleen, lymph nodes, and gut for up to 56-days following intramuscular injection of NRPV 19. The perfusion of alveolar macrophages in lung, Kupffer cells in liver, and marginal zone macrophages in spleen likely underlie the biodistribution of EuS and NRPV. Although EuS were observed in lower quantities within lymph nodes as compared to NRPV, EuS is still likely to be present in the splenic periarteriolar lymphoid sheath (PALS) that is rich in CD4+ T cells 44. High density ^177^Lu signal emanating from the spleen at day 5 and T2-weighted contrast at day 30 may also indicate that marginal zone- and red-pulp macrophages readily engulf EuS nanoprobes. In summary, EuS nanoprobes whose size and shape approximates that of NRPV display similar trafficking to spleen.

Although similarities were found between NRPV and EuS accumulation in spleen, liver and lung it is worth noting significant biodistribution disparities exist in other organs. Prior studies have demonstrated high RPV levels in kidney and gut exceeding those of spleen and liver 56 days after intramuscular dosing with poloxamer-encapsulated NRPV 19. By contrast, intravenous co-dosing of bismuth-sulfate nanorods (BSNRs) with NRPV favored drug deposition in spleen and lymph node rather than kidney by four weeks post-injection 15. Our findings recapitulate the notion that IV administration of NRPV enhances deposition in reticuloendothelial organs rather than those that facilitate drug excretion. Differences in particle biodistribution in non-lymphoid organs likely results from NRPV being nanoformulated with a poloxamer surfactant whereas EuS nanoprobes are encapsulated in cholesterol-based lipids. Future experiments will ensure that nanotheranostic compositions and route of administration is synchronized with those of nanoformulated antiretrovirals.

EuS nanoprobe biotracking reveals three therapeutic parameters. *First*, nanoparticles should be made with long-axis diameters below ~ 350 nm. This will serve to ensure that any defined therapeutics do not become entrapped in pulmonary capillaries that not only blunt systemic dissemination but could pose risk of pulmonary and cardiac failure. On balance, nanoparticles size must exceed 24 nm to avert inducing disseminated intravascular coagulation 45. *Second*, lipid components present in the particle's protective shell, core or complexed with therapeutic payloads should be of sufficient hydrophobicity to permeate lymph nodes. Reaching lymph nodes, where latently-infected memory CD4+ T cells are predominantly located, is critical for any therapeutic strategy developed to sterilize virus in an infected host 46. *Third*, any newly designed theranostic particles must mirror the physicochemical characteristics of antiretroviral prodrug nanocrystals, which show enhanced cell uptake and the slow ester hydrolysis of hydrocarbon promoieties, to parallel biodistribution patterns that now enable 90% inhibitory concentrations in plasma from months to up to one year 8.

Future investigations are planned to facilitate bench to bedside use of the EuS nanoprobes. First, intramuscular injection of EuS is required to simulate the route of long-acting antiretroviral administrations. Second, the utility of europium-153 fluorescence by confocal microscopy and flow cytometry must be employed to assess EuS uptake in divergent cell types. Third, serum enzyme profiles and histological assessments will need to be tested in order to determine potential EuS toxicity. Finally, the DSPE-PEG2000 lipid shell can be complexed to targeting ligands such as folic acid and tuftsin to evaluate whether surface modification improves particle delivery to lymphoid organs and the brain.

## Conclusions

EuS nanoprobes retain multimodal molecular imaging potential for delivery of rod-shaped therapeutic nanomaterials to HIV-1 reservoir sites. Advantages to this composition include reduced requirement for transition metals in particle synthesis, which confers improved biocompatibility to EuS and lowers production cost. Taken together, this technology provides a platform to accelerate the pace of nanoformulated ART development and maximize delivery of therapeutics to viral cell and tissue reservoirs.

## Supplementary Material

Supplementary materials.

## Abbreviations

^177^LuEuS: intrinsically doped lutetium-177 europium sulfide
AIDS: acquired immunodeficiency syndrome
ART: antiretroviral therapy
ATV: atazanavir
BSNR: bismuth sulfide nanorods
CAB: cabotegravir
Cas9: CRISPR-associated protein endonuclease 9
CPMG: Carr Purcell Meiboom Gill sequence
CRISPR: clustered regularly interspersed short palindromic repeats
EDX: energy dispersive X-ray spectroscopy
Eu: europium
EuCF: europium cobalt-ferrite
Eu-OA-OAM: europium-oleic acid-oleylamine
EuS: europium sulfide nanoprobes
FOV: field of view
HAADF-STEM: high-angle annular dark-field scanning transmission electron microscopy
HIV-1: human immunodeficiency virus type one
HR-TEM: high resolution transmission electron microscopy
IC90: 90% effective concentration
ICP-MS: inductively coupled plasma mass spectrometry
ID: injected dose
INSTI: integrase strand transfer inhibitor
IV: intravenous
M-CSF: macrophage colony stimulating factor
MDM: monocyte-derived macrophages
MP: mononuclear phagocytes
MRI: magnetic resonance imaging
NNRTI: non-nucleoside reverse transcriptase inhibitor
NRPV: nanoformulated rilpivirine
NRTI: nucleoside reverse transcriptase inhibitor
PBS: phosphate buffered saline
PI: protease inhibitor
ppm: parts per million
PrEP: pre-exposure prophylaxis
ROI: region of interest
RPV: rilpivirine
S: sulfide
SPECT-CT: single photon emission computed tomography overlaid computed tomography
TEM: transmission electron microscopy
UPLC: ultraperformance liquid chromatography
XRD: X-ray diffraction
μCi: microcuries
