# Supplementary material for: Europium sulfide nanoprobes predict antiretroviral drug delivery into HIV-1 cell and tissue reservoirs
Source: Nanotheranostics. 2021 Apr 27;5(4):417–30. doi: 10.7150/ntno.59568 (PMC8100756; doi:10.7150/ntno.59568)
Supplement: Supplementary file 1 — Supplementary materials. [file ntnov05p0417s1.pdf]

Supplementary material

Europium sulfide nanoprobes predict antiretroviral drug delivery into HIV-1 cell and tissue reservoirs

Jonathan Herskovitz[1], Mahmudul Hasan[2,3], Jatin Machhi[2], Insiya Mukadam[3], Brendan M. Ottemann[4], James R. Hilaire[2], Christopher Woldstad[5], JoEllyn McMillan[2], Yutong Liu[6], Javier Seravalli[7], Anandakumar Sarella[8], Howard E. Gendelman[2,3], and Bhavesh D. Kevadiya[2‡]

[1]*Department of Pathology and Microbiology, College of Medicine, University of Nebraska Medical Center, Omaha, NE 68198, USA*

[2]*Department of Pharmacology and Experimental Neuroscience, University of Nebraska Medical Center, Omaha, NE 68198 USA*

[3]*Department of Pharmaceutical Sciences, University of Nebraska Medical Center, Omaha, NE 68198 USA*

[4]*Department of Otorhinolaryngology, University of Kansas Medical Center, Kansas City, KS 66213 USA*

[5]*Brain Health Imaging Institute, Weill Cornell Medicine, New York, NY 10065 USA*

[6]*Department of Radiology, University of Nebraska Medical Center, Omaha, NE 68198 USA*

[7]*Department of Biochemistry, University of Nebraska Lincoln, Lincoln, NE 68588 USA*

[8]*Nebraska Center for Materials and Nanoscience, University of Nebraska Lincoln, Lincoln, NE 68588 USA*

[‡]Corresponding Author: Bhavesh D. Kevadiya, Assistant Professor, HIV Theranostics Laboratory, Department of Pharmacology and Experimental Neuroscience, Durham Research Center I 3030, University of Nebraska Medical Center, Omaha, NE 68198-5800; email: bhaveshd.kevadiya@unmc.edu; phone: 402 552 7617; Fax: 402 559 7495 (for review and correspondence)

Rilpivirine (RPV) quantitation by UPLC-UV/Vis

RPV was detected using a Waters ACQUITY ultra-performance liquid chromatography (UPLC) H-Class system with TUV detector and Empower 3 software (Milford, MA). RPV samples were separated on a Phenomenex Kinetex 5 µm C18 column (150 × 4.6 mm) (Torrance, CA). RPV was detected at 285 nm, using a mobile phase consisting of 65% 50 mM KH_2PO_4, pH 3.2, and 35% ACN and a flow rate of 1.0 mL/min. Drug content was determined relative to peak areas from standards (0.05–50 µg/mL) in MeOH.

Rilpivirine quantitation by UPLC-MS/MS

UPLC-MS/MS drug quantitation was performed in accordance with existing an laboratory protocol [1] on tissues from mice dosed with (^{177}Lu)EuS plus RPV (45 mg / kg) that were sacrificed 5 and 30 days post-treatment. Drug concentrations in tissue were determined by UPLC-MS/MS using a Waters Acquity UPLC-Xevo TQ-S micro mass spectrometry system (Milford, MA). RPV was separated using an AQUITY UPLC-BEH shield RP18 column (1.7 µm, 2.1 mm × 100 mm) with a 7 min gradient mobile phase consisting of A (7.5 mM ammonium bicarbonate in Optima-grade H2O adjusted to pH 7 using acetic acid) and B (100% Optima-grade MeOH) at a flow rate of 0.25 mL/min. Mobile phase B remained at 70% for the initial 4.75 min, followed by an increase to 95% B in 0.25 min and held constant for 0.75 min. Mobile phase B was reset to 70% in 0.25 min and the column equilibrated for 1.0 min before the next injection. A cone voltage of 92 volts and collision energy of 56 volts were used to detect RPV. Multiple reaction monitoring (MRM) transition 367.032 > 127.859 m/z was used for RPV quantification. Internal standards (IS; indinavir (IDV) 250 ng/mL; lopinavir (LPV) 500 ng/mL) was monitored at MRM transition 614.14 > 97.023 and 629.18 > 155.03 m/z respectively. Tissue analysis required between 10–200 mg of tissue (spleen, lymph node, liver, gut, lung, kidney, and brain) were diluted with 90% MeOH and homogenized. Tissue homogenates were mixed with MeOH

containing internal standard and vortexed for 3 min, followed by centrifugation at 17,000 g for 10 min. Supernatants were collected and mixed with Optima-grade H2O for UPLC-MS/MS analysis. Tissue standards were extracted at a final concentration of 0.5–2500 ng/mL.

Europium-153 quantitation by ICP-MS

Europium and sulfur quantifications were performed by inductively coupled plasma mass spectrometry (ICP-MS) at the University of Nebraska-Lincoln's Spectroscopy and Biophysics Core Facility, using an Agilent Model 7500cx (Santa Clara, CA, USA) coupled with a 96-well plate autosampler Model SC/DX4 from Elemental Scientific, Inc. (Omaha, NE, USA), operating in Mix-Gas collision/reaction mode (3.5 mL H2 and 1.5 mL He per minute). Other conditions were: plasma power, 1500 W; carrier gas flow, 1 L/minute; makeup gas flow, 0.15 L/minute; sample depth, 8 mm; plasma gas, 15 L/minute. The concentrations were calculated against an external calibration curve with 50 μg/L of Ga used as internal standard (IS) throughout (71Ga isotope). Tissue samples were suspended in 4 times the volume of metal-grade nitric acid, incubated at room temperature for up to 2 hours followed by overnight digestion at 65°C. The samples were cooled and diluted 20-fold into the autosampler at a final concentration of 10 mg/mL. The concentrations were calculated using an external calibration curve prepared from ICP-MS standards from Inorganic Ventures (Christiansburg, VA, USA).

REFERENCES

1. Hilaire JR, Bade AN, Sillman B, Gautam N, Herskovitz J, Dyavar Shetty BL, et al. Creation of a long-acting rilpivirine prodrug nanoformulation. J Control Release. 2019; 311-312: 201-11.
